# Facial Discoid Dermatosis Imaging with Line-Field Confocal Optical Coherence Tomography and Reflectance Confocal Microscopy—A Case Report and Literature Review

**DOI:** 10.3390/jpm16070360

**Published:** 2026-07-01

**Authors:** Joanna Zygadło, Leszek Blicharz, Joanna Czuwara, Joanna Nowaczyk, Karolina Makowska, Małgorzata Olszewska, Lidia Rudnicka

**Affiliations:** 1 Department of Dermatology, Medical University of Warsaw, Nowogrodzka 59, 02-006 Warsaw, Poland; 2 Department of Dermatology, University Medical Center of Johannes Gutenberg University Mainz, 55131 Mainz, Germany

**Keywords:** facial discoid dermatosis, line-field confocal optical coherence tomography, reflectance confocal microscopy, non-invasive skin imaging, pityriasis rubra pilaris, dermatopathology, personalized medicine

## Abstract

**Background/Objectives**: Facial discoid dermatosis is a rare inflammatory dermatosis presenting with round, superficial erythematous lesions located on the face. Diagnosis may be challenging and often requires careful clinicopathological correlation due to overlapping clinical and histopathological features. Skin lesions are typically resistant to a wide range of topical and systemic treatments. From the perspective of personalized medicine, improved phenotyping of rare inflammatory dermatoses may support more precise diagnosis, individualized therapeutic decisions, and non-invasive disease monitoring. This study aimed to characterize facial discoid dermatosis using line-field confocal optical coherence tomography and reflectance confocal microscopy and to discuss its differential diagnosis and therapeutic implications. **Methods**: We report a case of facial discoid dermatosis in a 35-year-old patient examined with line-field confocal optical coherence tomography and reflectance confocal microscopy. The imaging findings were interpreted in correlation with clinical and histopathological features. A literature review was performed to summarize differential diagnoses, therapeutic perspectives, and the proposed relationship between facial discoid dermatosis and pityriasis rubra pilaris. **Results**: Non-invasive imaging revealed morphological features consistent with a psoriasiform inflammatory dermatosis and provided additional in vivo information supporting the diagnosis. The literature review showed limited evidence for a direct association between facial discoid dermatosis and pityriasis rubra pilaris, with only isolated reports suggesting possible overlap or progression. **Conclusions**: Facial discoid dermatosis appears to represent a distinct psoriasiform dermatosis. Line-field confocal optical coherence tomography and reflectance confocal microscopy may contribute to a personalized diagnostic approach by supporting differential diagnosis and potentially guiding individualized monitoring in rare inflammatory facial dermatoses.

## 1. Introduction

Facial discoid dermatosis (FDD) is a relatively recently described entity, first reported by Ko et al. in 2010 [1]. To date, FDD remains a rarely recognized condition [2,3]. The available literature on FDD is limited to case reports and small case series. Therefore, its true incidence, prevalence, and geographic distribution remain unknown. The pathogenesis has not been established, although the histopathological findings suggest a chronic inflammatory papulosquamous process affecting the face.

Clinically, FDD presents as discrete, discoid, well-demarcated, pink-to-orange papulosquamous facial lesions, which show resistance to topical therapies and usually remain stable over time [1,4]. The lesions measure from a few millimeters to 3.5 cm [1] and favor the cheeks (93%), chin (69%), and forehead (38%) [5]. Itching and annular scale may be present [6]. The age of onset is usually between the second and fourth decades of life [1]. FDD shows a strong female predominance (5:1) [2,5,7,8]. Histopathological features may include hyperkeratosis, focal parakeratosis, acanthosis with psoriasiform hyperplasia, spongiosis, follicular plugging, and involuted sebaceous lobules [1,6,9,10].

Non-invasive skin imaging with line-field confocal optical coherence tomography (LC-OCT) and reflectance confocal microscopy (RCM) enable real-time microscopic assessment of skin lesions without biopsy. This is particularly useful in rare, treatment-refractory facial dermatoses such as FDD, where repeated biopsies of cosmetically sensitive areas may lead to scarring and patient discomfort. By providing in vivo morphological information at the level of individual lesions, these techniques support a personalized approach to diagnosis and longitudinal monitoring in such challenging cases.

## 2. Methods

### 2.1. Case

The case described in this report was managed at the Department of Dermatology, Medical University of Warsaw. Dermoscopy was performed with a DermLite DL4 dermatoscope, LC-OCT with the deepLive™ device (DAMAE Medical, Paris, France) in both vertical and horizontal acquisition modes, and RCM with the VivaScope^®^ 1500/3000 (Caliber I.D., Rochester, NY, USA). Histopathological sections were stained with hematoxylin and eosin and reviewed by a board-certified dermatopathologist (J.C.).

### 2.2. Literature Search and Selection

A narrative literature review was conducted to summarize the current evidence on facial discoid dermatosis, with particular focus on (i) its clinical, dermoscopic, histopathological and in vivo imaging features, (ii) its differential diagnosis, (iii) reported therapeutic strategies, and (iv) the proposed relationship between FDD and pityriasis rubra pilaris (PRP). The following electronic databases were searched from inception to March 2026: PubMed/MEDLINE, Embase, Scopus and Web of Science using the term: “facial discoid dermatosis”. Peer-reviewed publications in English were included. Records were screened by 4 authors (J.Z., L.B., J.N. and K.M.) in two stages: titles and abstracts were first reviewed to identify thematically relevant publications, and full-text assessment was subsequently performed only for those records judged relevant to the scope of the review. Owing to the rarity of FDD, the predominance of single-patient case reports and small case series in the literature, a formal quality appraisal of individual studies was not performed, and evidence was synthesized narratively, consistent with the descriptive aim of this review.

## 3. Case Presentation

**Figure 1:**
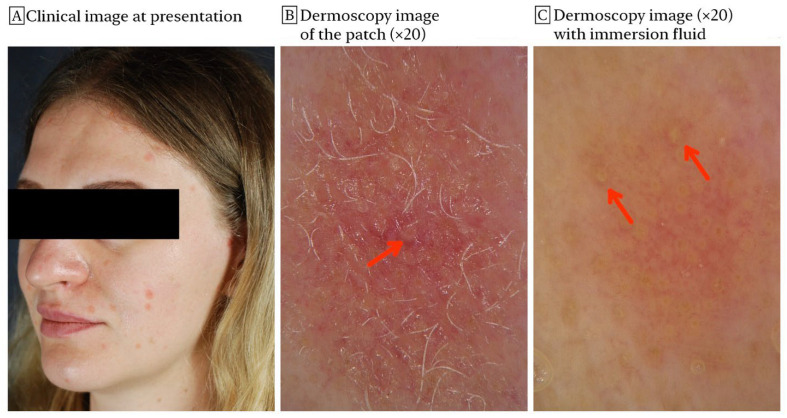
(**A**) Clinical photograph of a 35-year-old patient with facial discoid dermatosis. (**B**,**C**) Dermoscopy of skin lesions showed large yellow dots corresponding to enlarged follicular ostia (red arrows).

A 35-year-old woman presented with round, erythematous, scaly patches located on the cheeks, forehead, and chin (Figure 1A). The lesions measured up to 1.5 cm in diameter and had slightly progressed over two years. History revealed no inciting factors. At the time of onset, histopathological examination performed outside of our institution demonstrated hyperkeratosis, focal parakeratosis, and mild spongiosis. No conclusive diagnosis was made. Prior to presenting at our center, the patient had received treatment with systemic doxycycline and numerous topical agents including tacrolimus ointment, imiquimod cream, diflucortolone/isoconazole cream and adapalene gel with no improvement. She had no concomitant diseases, allergies and was not receiving any chronic medication.

**Figure 2:**
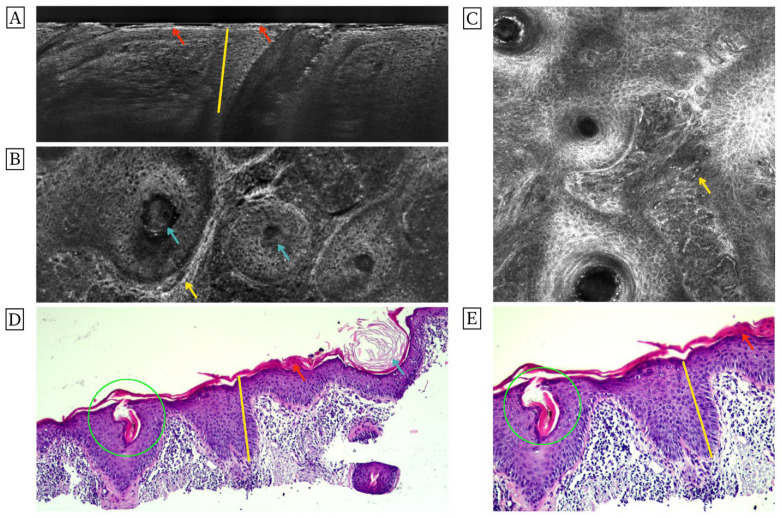
(**A**,**B**) Images from line-field confocal optical coherence tomography in vertical and horizontal modes of acquisition and (**C**), reflectance confocal microscopy. The primary findings involved epidermal acanthosis (yellow vertical lines), focal parakeratosis (red arrows), follicular plugging (blue arrows), and dilated blood vessels in the papillary dermis surrounded by a dense inflammatory infiltrate (yellow arrow). (**D**,**E**) Histopathology confirmed those findings and additionally demonstrated features of demodicosis (green circle) (H&E; original magnification ×40 in (**D**) and ×100 in (**E**)).

Dermoscopy showed fine scale overlying dilated blood vessels and large yellow dots corresponding to enlarged follicular ostia (Figure 1B), which were better seen after the application of immersion fluid (Figure 1C). We performed line-field confocal optical coherence tomography in two modes of acquisition: vertical and horizontal (Figure 2A,B), and reflectance confocal microscopy (Figure 2C). Both LC-OCT and RCM revealed acanthosis, focal parakeratosis (hyperreflective particles in the keratinocytes in the stratum corneum), areas of fine scale overlying the epidermis (hyperreflective superficial streaks), and plugs in dilated follicular openings (hyperreflective signal in the follicular ostia). In the papillary dermis, hyporeflective linear areas corresponding to dilated blood vessels were seen. They were surrounded by round hyperreflective cells consistent with an inflammatory infiltrate. To confirm the non-invasive imaging findings in histopathology, a shave biopsy was performed (Figure 2D,E). The histopathological features were analogous to those observed in LC-OCT and RCM. The epidermis showed parakeratosis occasionally alternating with orthokeratosis. Histopathology confirmed a predominantly lymphohistiocytic infiltrate. Additionally, Demodex mites were observed within hair follicles and were interpreted as a concomitant finding. Direct and indirect immunofluorescence tests were negative. Laboratory results showed no abnormalities.

Based on the clinical features, recalcitrant disease course and histological findings, the diagnosis of facial discoid dermatosis was made. The patient was prescribed oral hydroxychloroquine 200 mg twice daily, topical ivermectin cream and tacrolimus ointment. After 3 months, she showed only minor improvement.

## 4. Discussion

To the best of our knowledge, this is the first report presenting LC-OCT and RCM features of FDD. Both methods enable precise, real-time non-invasive imaging of the epidermis and superficial dermis. RCM is superior to LC-OCT in terms of resolution, but inferior with respect to the depth of analysis [11,12]. Additionally, LC-OCT enables both vertical and horizontal imaging, which yields the possibility of creating a three-dimensional model of the analyzed skin sample. Taken together, these methods can be regarded as complementary tools in lesions occupying not only the epidermis, but also the superficial dermis. Indeed, in FDD, which is a superficial inflammatory process, we showed a close correlation between histopathology and both LC-OCT and RCM findings. This underscores the applicability of these tools in differentiating between FDD and other possible diagnoses, such as psoriasis, pityriasis rubra pilaris (PRP), seborrheic dermatitis, discoid lupus erythematosus, Senear–Usher syndrome, and tinea faciei (Table 1).

### 4.1. Facial Discoid Dermatosis—A Distinct Psoriasiform Dermatosis

The clinical picture and histology of FDD favor its classification as a psoriasiform dermatosis sharing many features with PRP. Gan et al. [24] reported the development of type II PRP years after the initial diagnosis of FDD and proposed classifying it as a novel type VII PRP, following the types I–V originally distinguished by Griffiths [25] and type VI proposed by Miralles et al. [26] in HIV-infected individuals. However, only one out of eight patients in the described case series showed disease progression to type II PRP [24], and no similar case reports have been published to date. Furthermore, Allegue et al. [9] described a case of unilateral ectropion in the course of FDD involving the lower eyelid, which also prompted them to associate this disease with PRP. This assumption was based on the well-established risk of ectropion occurrence in a subset of patients with facial PRP lesions [27]. The treatment of FDD led to the resolution of ocular symptoms. However, in our opinion, this remote association is insufficient to justify the identification of FDD as a subtype of PRP. Moreover, FDD does not typically display the hallmark features of PRP such as palmoplantar keratoderma or classic “islands of sparing” [28]. The genetic background of FDD is still unknown. Mutations in CARD14, a gene linked to PRP and related papulosquamous eruptions [29], have not been reported in FDD cases; thus, a shared molecular basis cannot be ruled out, and dedicated genetic studies are warranted.

Therefore, we propose that the chronic, localized, treatment-refractory nature of FDD and its distinct demographic and clinical pattern argue for its recognition as an independent entity within the spectrum of psoriasiform dermatoses rather than as a PRP variant.

### 4.2. Treatment

FDD is resistant to a wide range of conventional treatments, which constitutes one of its most distinctive hallmarks [1]. Phototherapy may be considered, in theory, due to the psoriasiform inflammatory pattern of FDD. However, the available evidence does not support its efficacy. Rahmatulla et al. and Bohdanowicz and DeKoven reported the use of narrowband UVB phototherapy in a total of three patients with FDD, without clinical improvement [4,7]. Topical agents such as corticosteroids, retinoids, antifungals, calcipotriol, calcineurin inhibitors, and imiquimod, as well as other treatment modalities, including oral acitretin, doxycycline, prednisone, methotrexate, hydroxychloroquine, and pulsed-dye laser, have been reported in the literature [1,9,30]. One study [30] reported complete response with topical rapamycin (sirolimus) 0.2% after one-month therapy in a patient recalcitrant to topical treatments (corticosteroids, tacrolimus, pimecrolimus, crisaborole) and systemic therapy with secukinumab and guselkumab.

Bohdanowicz and DeKoven [7] published a paper demonstrating partial resolution of skin lesions after a two-month therapy with calcipotriol/betamethasone ointment combined with low-dose acitretin. Amarnani et al. [31] reported an almost complete remission of FDD with topical clobetasol propionate and calcipotriol for three weeks followed by topical calcipotriol monotherapy for another three weeks. Favorable response to treatment prompted the authors to advocate the use of topical calcipotriol instead of systemic medications and long-term topical steroid therapy to avoid treatment-related adverse events. This is reinforced by the already mentioned case of Allegue et al. [9], who also achieved a remission of FDD lesions following a two-month therapy with daily application of calcipotriol/betamethasone ointment. Nevertheless, maintenance with topical 0.1% tacrolimus twice weekly was necessary to preserve disease remission. Finally, Rypka et al. [32] and Fera et al. [8] reported significant improvement of FDD following treatment with ustekinumab. A similar response to ustekinumab has been reported in CARD14-associated papulosquamous eruptions of the face, which share features of both psoriasis and PRP [29]. Whether ustekinumab works in FDD through the same IL-12/23/Th17 pathway or through a still-unrecognized role of CARD14 needs further genetic studies.

Our patient received numerous topical and systemic treatments with little effect. Partial improvement achieved at our center most probably resulted from the suppression of Demodex spp.-induced inflammation by topical ivermectin. The patient refused to apply the calcipotriol/betamethasone preparation for fear of potential side effects following its application on the face.

From a personalized medicine perspective, LC-OCT and RCM may support a more individualized assessment of rare facial dermatoses such as FDD. These methods provide real-time microscopic information without repeated biopsies, which is particularly important for lesions located on the face. They may help refine the differential diagnosis, guide treatment decisions, and monitor the response to therapy in a non-invasive way.

## 5. Conclusions

FDD is a rare skin condition typically affecting adult females. It is a disfiguring disease characterized by a chronic course and frequent resistance to conventional treatments. FDD seems to belong to the spectrum of psoriasiform dermatoses, which is supported by its clinical and histological picture. Although progression to PRP has been reported in one case described to date, the classification of FDD as a subtype of PRP is questionable. As a superficial inflammatory process, it can be diagnosed using LC-OCT, RCM, or a combination of both. Based on the available data, it seems that patients could benefit most from the use of topicals combining calcipotriol and betamethasone, as well as from ustekinumab and other biologics targeting the IL-12/23 and IL-17 pathways. This case illustrates how non-invasive imaging may contribute to personalized dermatology by supporting diagnosis and treatment selection in rare, recalcitrant inflammatory dermatoses of the face. Future multicenter, prospective, and molecular studies are needed to validate LC-OCT and RCM findings, standardize diagnostic criteria, and better define targeted therapeutic strategies for patients with FDD.

## Figures and Tables

**Table 1:** Differential diagnoses of facial discoid dermatosis.

Disease	Differentiating Clinical and Laboratory Features	Dermoscopy	Line-Field Confocal Optical Coherence Tomography	Reflectance Confocal Microscopy	Histopathology
Facial discoid dermatosis [1,4,5]	• Discoid, well-demarcated hyperkeratotic lesions confined to the face • Female predominance • Treatment resistant	• Large yellow dots corresponding to enlarged follicular ostia • Dilated blood vessels • Fine scale	• Acanthosis • Focal parakeratosis (hyperreflective nucleated cells in stratum corneum) • Areas of fine scale overlying the epidermis (hyperreflective superficial streaks) • Plugs in dilated follicular openings (hyperreflective signal in the follicular ostia) • Hyporeflective linear areas corresponding to dilated blood vessels in the papillary dermis • Dense perivascular inflammatory infiltrate (round hyperreflective cells) *		• Hyperkeratosis • Focal parakeratosis occasionally alternating with orthokeratosis • Acanthosis with psoriasiform hyperplasia • Follicular plugging • Possible spongiosis and involuted sebaceous lobules
Pityriasis rubra pilaris [13,14,15]	• Hyperkeratotic follicular papules coalescing into orange-red scaly plaques • Islands of sparing • Palmoplantar keratoderma • Response to oral retinoids and biologics	• Round or oval yellow patches surrounded by linear dotted vessels • Keratin plugs • In erythroderma: orange blotches, islets of sparing with reticular vessels	• Not reported	• Foci of parakeratosis intermingled with areas of orthokeratosis (cells with bright borders and dark centers) • Parakeratosis around hair follicles • Preserved stratum granulosum and stratum spinosum • Reduced number of dermal papillae (open black roundish structures) showing irregular shape and size • Inflammatory cells inside enlarged blood vessels	• Alternating areas of ortho- and parakeratosis (checkerboard pattern) • Focal or confluent hypergranulosis • Irregular acanthosis in the form of short and broad rete ridges • Thick supra-papillary plates • Sparse dermal superficial perivascular lymphohistiocytic infiltrate • Follicular plugging with parakeratosis at the edges of the follicular orifice (‘shoulder parakeratosis’) • Absence of neutrophilic exocytosis and intraepidermal neutrophilic microabscesses
Psoriasis [13,16,17]	• Common extrafacial involvement • Koebner phenomenon • Possible nail dystrophy and arthritis • Improvement on topical anti-inflammatory treatments	• White scale • Dotted vessels in a diffuse distribution	• Thickening of the viable epidermis with elongation of the rete ridges • Hyporeflective, elongated dermal papillae containing dark canalicular structures (ectatic vessels)	• Parakeratosis • Thinning or absence of the stratum granulosum • Munro microabscesses (clusters of bright cells in the epidermis) • Inflammatory cells in the epidermis • Non-edged dermal papillae • Dilated blood vessels in the papillary dermis	• Confluent parakeratosis, Munro microabscesses and spongiform pustules of Kogoj • Diminished or absent stratum granulosum; suprapapillary thinning • Elongation of the dermal papillae and rete ridges • Dilation of the blood vessels in the papillary dermis • Mixed perivascular infiltrate with neutrophil exocytosis into the epidermis
Seborrheic dermatitis [13,18]	• Most severe in sebum-rich areas • Periods of remissions and exacerbations • Good response to antifungals and anti-inflammatory agents	• Yellow scale • Dotted vessels in a patchy distribution	• Not reported	• Spongiosis (hyporeflective areas at the level of the epidermis with broadband intercellular spaces), • Dermal inflammation and horizontally oriented dilated blood vessels	• Spongiosis • Mounds of (so-called ‘shoulder’) parakeratosis at the edges of follicular ostia, often with neutrophils • Scale crust overlying the epidermis • Chronic lesions may show signs of psoriasiform hyperplasia
Discoid lupus erythematosus [13,17,19]	• Photosensitivity • May be associated with scarring and cicatricial alopecia • May coexist with symptoms of SLE • Good response to antimalarials • Granular IgG/IgM/C3 deposits at the dermo-epidermal junction in direct immunofluorescence	• Follicular plugging • White scale, particularly at the periphery • Possible branching vessels Possible white structureless areas (scarring) • Perifollicular whitish halos • Brown-gray dots/structures	• Thickening and disruption of the entrance signal (hyperkeratosis) • Thinned layer below the entrance signals (epidermal atrophy) • Patchy reduction in reflectivity in the upper dermis (lichenoid infiltrates and edema of the upper dermis) • Dilated vessels in the upper dermis	• Hyperkeratosis (also in the adnexal infundibula), epidermal disarray, interface dermatitis (hyperreflective inflammatory cells obscuring the normal ring formation of the basal cells around the dermal papillae) • Dilated blood vessels • Periadnexal and perivascular inflammatory infiltrate	• Interface dermatitis, periadnexal and perivascular lymphohistiocytic infiltrates • Thickening of the basement membrane, degeneration of the basal layer • Follicular plugging • Dermal mucin depositions
Senear–Usher syndrome (pemphigus erythematosus) [20]	• Photosensitivity • Positive direct immunofluorescence in most patients (simultaneous intercellular and dermo-epidermal junction staining) • Antinuclear antibodies positive in 30–80% of patients • Possible symptoms of SLE	• Not reported	• Not reported	• Not reported	• Subcorneal acantholysis • Acanthosis with hyperkeratosis, follicular accentuation • Occasionally interface dermatitis with basal layer degeneration
Tinea faciei [13,21]	• Peripheral scaly rim with central clearing (annular distribution) • Relatively quick, centrifugal progression • Itch • Positive fungal culture • Good response to antifungals	• Diffuse erythema • Dotted vessels at the periphery • Peripheral scale • Broken hairs • Possible perifollicular pustules	• Not reported	• Mycelium (bright linear structures in the stratum corneum) • Blister: round hyporeflective structure localized in the stratum corneum • Inflammatory infiltrate: hyperreflective polymorphonuclear cells, accompanied by light flashes located in the stratum corneum or the upper layer of acanthosis	• Spongiosis, parakeratosis, acanthosis, accumulation of neutrophils in the epidermis • Possible dermal edema and chronic inflammatory infiltrates • PAS demonstrates fungal hyphae
Cutaneous sarcoidosis [22,23]	• In up to 35% of the chronic sarcoidosis cases • Polymorphic lesions that may imitate other skin diseases • The most common variant is maculopapular lesions	• Diverse manifestations • Multiple linear and branching vessels • Yellowish-orange globular and leaf-like structures • Depigmented scar-like areas	• Subepidermal hyporeflective ovoid formations, surrounded by bright connective tissue	• Hyporeflective ovoid nodular structures • Increased vascularization • Bright beaded-like structures and enlarged hyperreflective cells within each individual nodule surrounded by a bright stroma and accompanied by smaller hyperreflective cells	• Noncaseating subepidermal granulomas (conglomerates of epithelioid histiocytes, giant cells, and macrophages)

* For facial discoid dermatosis, line-field confocal optical coherence tomography and reflectance confocal microscopy findings are presented in a single merged cell because both methods revealed features in the case described. LC-OCT—line-field confocal optical coherence tomography; RCM—reflectance confocal microscopy; SLE—systemic lupus erythematosus; PAS—periodic acid–Schiff.

## Data Availability

All further inquiries should be directed to the corresponding author.

## Abbreviations

The following abbreviations are used in this manuscript:

FDD	Facial discoid dermatosis
LC-OCT	Line-field confocal optical coherence tomography
RCM	Reflectance confocal microscopy
PRP	Pityriasis rubra pilaris
SLE	Systemic lupus erythematosus
PAS	Periodic acid–Schiff
H&E	Hematoxylin and eosin
IL-17	Interleukin-17
IL-23	Interleukin-23
CARD14	Caspase recruitment domain family member 14
HIV	Human immunodeficiency virus
IgG	immunoglobulin G
IgM	immunoglobulin M
C3	Complement component 3

## References

[1] Ko C.J., Heald P., Antaya R.J., Bolognia J.L. (2010). Facial discoid dermatosis. Int. J. Dermatol..

[2] Butrón-Bris B., Buján C., Berenguer-Ruiz S., Martos-Cabrera L., Rodríguez-Jiménez P., Fraga J., Eguren C., Serrano-Pardo R., Iranzo P., Mascaró J.M. (2025). [Translated article] Facial Discoid Dermatosis. A 13-Case Series. Actas Dermo-Sifiliográficas.

[3] Rodríguez-Troncoso M., De la Torre-Gomar F.J., Pegalajar-García M.D., Martín-Castro A., Ruiz-Villaverde R. (2025). Oral Isotretinoin for Facial Discoid Dermatosis: Effectiveness, Safety, and Clinical Suitability—Case Report of Complete Response and Literature Review. Case Rep. Dermatol..

[4] Rahmatulla S., Batta K., Tatnall F., Sandhu D., Brown V. (2021). Facial discoid dermatosis: A cosmetically disfiguring and challenging condition to treat. Skin Health Dis..

[5] Condal L., Quer A., Ferrándiz C., Bielsa I. (2021). Facial Discoid Dermatosis: An Enigmatic Disease. Actas Dermosifiliogr..

[6] Salman A., Tekin B., Berenjian A., Cinel L., Demirkesen C. (2015). Facial discoid dermatosis: A further case of a novel entity. J. Dermatol..

[7] Bohdanowicz M., DeKoven J.G. (2018). Improvement in facial discoid dermatosis with calcipotriol/betamethasone ointment and low-dose acitretin. Clin. Exp. Dermatol..

[8] Fera C., Chamaillard M., Beylot-Barry M., Levavasseur M. (2025). Facial Discoid Dermatosis, a Still Unknown Entity. Acta Derm. Venereol..

[9] Allegue F., Fachal C., Iglesias B., Zulaica A. (2022). Facial Discoid Dermatosis: A New Variant of Pityriasis Rubra Pilaris?. Actas Dermosifiliogr..

[10] Welborn M., Fletcher D., Motaparthi K. (2022). Atrophy of sebaceous lobules in facial discoid dermatosis: A link to psoriasis and seborrheic dermatitis?. J. Cutan. Pathol..

[11] Welzel J., Schuh S. (2017). Noninvasive diagnosis in dermatology. J. Dtsch. Dermatol. Ges..

[12] Ogien J., Tavernier C., Fischman S., Dubois A. (2023). Line-field confocal optical coherence tomography (LC-OCT): Principles and practical use. Ital. J. Dermatol. Venerol..

[13] Errichetti E., Stinco G. (2016). Dermoscopy in General Dermatology: A Practical Overview. Dermatol. Ther..

[14] Wang D., Chong V.C., Chong W.S., Oon H.H. (2018). A Review on Pityriasis Rubra Pilaris. Am. J. Clin. Dermatol..

[15] Pietroleonardo L., Di Stefani A., Campione E., Chimenti S., Orlandi A., Bianchi L. (2013). Confocal reflectance microscopy in pityriasis rubra pilaris. J. Am. Acad. Dermatol..

[16] Verzì A.E., Broggi G., Micali G., Sorci F., Caltabiano R., Lacarrubba F. (2022). Line-field confocal optical coherence tomography of psoriasis, eczema and lichen planus: A case series with histopathological correlation. J. Eur. Acad. Dermatol. Venereol..

[17] Hoogedoorn L., Peppelman M., van de Kerkhof P.C., van Erp P.E., Gerritsen M.J. (2015). The value of in vivo reflectance confocal microscopy in the diagnosis and monitoring of inflammatory and infectious skin diseases: A systematic review. Br. J. Dermatol..

[18] Agozzino M., Berardesca E., Donadio C., Franceschini C., de Felice C.M., Cavallotti C., Sperduti I., Ardigò M. (2014). Reflectance confocal microscopy features of seborrheic dermatitis for plaque psoriasis differentiation. Dermatology.

[19] Gambichler T., Hyun J., Moussa G., Tomi N.S., Boms S., Altmeyer P., Hoffmann K., Kreuter A. (2007). Optical coherence tomography of cutaneous lupus erythematosus correlates with histopathology. Lupus.

[20] Hobbs L.K., Noland M.B., Raghavan S.S., Gru A.A. (2021). Pemphigus erythematosus: A case series from a tertiary academic center and literature review. J. Cutan. Pathol..

[21] Hui D., Sun X., Xu A. (2013). Evaluation of reflectance confocal microscopy in dermatophytosis. Mycoses.

[22] Thamm J.R., Welzel J., Schuh S. (2023). Line-field confocal optical coherence tomography, optical coherence tomography and reflectance confocal microscopy in a case of cutaneous sarcoidosis. J. Eur. Acad. Dermatol. Venereol..

[23] Segurado-Miravalles G., Fernández-Nieto D., Suárez-Valle A., Guevara B., Katherine E., Navarro A., González S. (2021). Reflectance confocal microscopy of cutaneous sarcoidosis. Skin. Res. Technol..

[24] Gan E.Y., Ng S.K., Goh C.L., Lee S.S.J. (2018). Recalcitrant psoriasiform dermatosis of the face: Is it related to pityriasis rubra pilaris?. J. Cutan. Pathol..

[25] Griffiths W.A. (1980). Pityriasis rubra pilaris. Clin. Exp. Dermatol..

[26] Miralles E.S., Núñez M., De Las Heras M.E., Pérez B., Moreno R., Ledo A. (1995). Pityriasis rubra pilaris and human immunodeficiency virus infection. Br. J. Dermatol..

[27] Haridas A.S., Sullivan T.J. (2016). Surgical Management of Cicatricial Ectropion in Pityriasis Rubra Pilaris. Ophthalmic Plast. Reconstr. Surg..

[28] Joshi T.P., Duvic M. (2024). Pityriasis Rubra Pilaris: An Updated Review of Clinical Presentation, Etiopathogenesis, and Treatment Options. Am. J. Clin. Dermatol..

[29] Craiglow B.G., Boyden L.M., Hu R., Virtanen M., Su J., Rodriguez G., McCarthy C., Luna P., Larralde M., Humphrey S. (2018). CARD14-associated papulosquamous eruption: A spectrum including features of psoriasis and pityriasis rubra pilaris. J. Am. Acad. Dermatol..

[30] Huang Y.W., Tsai T.F. (2024). Topical rapamycin (sirolimus) for the treatment of facial discoid dermatosis. Eur. J. Dermatol..

[31] Amarnani R., Hughes S., Morris-Jones R., Kanwar A.J., Bunker C.B. (2022). Persistent facial discoid dermatosis successfully treated with topical calcipotriol. Clin. Exp. Dermatol..

[32] Rypka K.J., Fulk T.S., Afsaneh A., Miller D.D., Goldfarb N.I. (2022). Improvement of Facial Discoid Dermatosis with Ustekinumab Treatment. JAMA Dermatol..

